# Effect of Potassium Supplementation on Endothelial Function: A Systematic Review and Meta-Analysis of Intervention Studies

**DOI:** 10.3390/nu15040853

**Published:** 2023-02-08

**Authors:** Lanfranco D’Elia, Francesco P. Cappuccio, Maria Masulli, Ersilia La Fata, Domenico Rendina, Ferruccio Galletti

**Affiliations:** 1 Department of Clinical Medicine and Surgery, “Federico II” University of Naples Medical School, 80138 Naples, Italy; 2 World Health Organization Collaborating Centre for Nutrition, Warwick Medical School, University of Warwick, Coventry CV4 7AL, UK; 3 Department of Medicine, University Hospital Coventry and Warwickshire NHS Trust, Coventry CV2 2DX, UK; 4 UOC Cure Domiciliari, ASL Napoli 2 Nord, 80078 Frattaminore, Italy

**Keywords:** dietary potassium, potassium intake, potassium consumption, endothelial function, flow-mediated dilation, meta-analysis

## Abstract

(1) Background: Endothelial dysfunction is an early predictor of cardiovascular diseases. Although a large body of evidence shows an inverse association between potassium intake and cardiovascular risk, the studies on endothelial function provided contrasting results. Thus, we carried out a systematic review and a meta-analysis of the available intervention studies of the potassium supplementation on endothelial function. (2) Methods: A systematic search of the online databases available (up to December 2022) was conducted including the intervention trials that reported flow-mediated dilation (FMD) changes—a non-invasive method of assessing endothelial function—after two different potassium intake regimens. For each study, the mean difference (MD) and 95% confidence intervals were pooled using a random effect model. (3) Results: Five studies met the pre-defined inclusion criteria and provided eight cohorts with 332 participants. In the pooled analysis, potassium supplementation was associated with a significant increase in FMD (MD: 0.74%), with a higher effect for a urinary potassium excretion higher than 90 mmol/day. There was a moderate heterogeneity among studies (I^2^ = 59%), explained by the different amount of potassium supplementation. (4) Conclusions: The results of our meta-analysis indicate that dietary potassium supplement improves endothelial function. This effect is directly associated with the amount of potassium supplement. The findings support the campaigns in favour of an increase in dietary potassium intake to reduce cardiovascular risk.

## 1. Introduction

Potassium is one of the principal minerals for human nutrition and it is involved in several physiological processes, especially blood pressure regulation. Several observational and intervention studies showed an inverse relationship between dietary potassium intake and blood pressure [1,2]. In addition, many clinical investigations found a positive effect of potassium-rich diet on cardiovascular health, in part independently of the effects on blood pressure [3,4,5].

In recognition of the compelling evidence of the benefits associated with higher potassium intake than is currently consumed, the World Health Organization has issued recommendations for a target daily dietary intake of at least 90 mmol of potassium per day in adults [6]. Several approaches may be adopted to achieve a minimum daily intake for potassium [7]: dietary modification (substituting potassium-low foods for fruits, vegetables, beans, and nuts) [8], use of potassium-enriched salt substitutes (to be used in place of sodium chloride salt) [9], and potassium supplementation with slow-release potassium salts, generally safe at a low dose [10].

Experimental data showed that a high potassium diet may exert a protective effect against the development of cardiovascular diseases. An increase in potassium intake was shown to increase the production of endothelial nitric oxide [11] and suppress that of reactive oxygen species [12,13,14]. Moreover, increased potassium concentration in cell cultures ameliorated vascular endothelium stiffness and increased nitric oxide release [15]. These data support the biological plausibility for an influence of dietary potassium intake on endothelial dysfunction, which is involved in the development of atherosclerosis and precedes structural vascular alterations [16].

Endothelial function can be evaluated by different non-invasive tests such as flow-mediated vasodilation (FMD), a widely used tool for clinical studies [16]. Impaired (i.e., reduced) FMD was associated with conditions predisposing to atherosclerosis and cardiovascular disease [16].

Despite experimental and observational results, intervention studies about the effect of potassium intake on endothelial function reported contrasting results [17,18,19,20,21,22]. Actually, some of the intervention studies had important methodological limitations such as low statistical power, heterogeneity of the participants’ and studies characteristics, or the use of mixed interventions. A meta-analysis of the intervention studies did not find any association between potassium supplementation and endothelial function [23]; however, this meta-analysis had, in turn, multiple limitations.

Considering new evidence currently available [20,22] and to provide stronger evidence of the role of supplementation of potassium intake on FMD as an expression of endothelial function, we carried out a systematic review and a meta-analysis of the available intervention studies meeting predefined inclusion criteria to test the hypothesis that potassium supplementation improves endothelial function, calculate the extent of the effect, and assess the effect of potential confounders on this relationship.

## 2. Materials and Methods

### 2.1. Data Sources and Search Strategy

This meta-analysis was conducted according to the PRISMA statement [24] (Supplemental Table S1). The study protocol was preregistered (CRD42022381314). A systematic search of the available publications was performed using WOS, Scopus, and MEDLINE/PubMed up to 1 December 2022. The search strategy—without restrictions—included the expressions “potassium excretion” OR “dietary potassium” OR “potassium intake” OR “potassium consumption” AND “endothelial damage” OR “endothelial function” OR “flow mediated dilation” OR “vascular function”, or combinations thereof, either in medical subject headings or in the title/abstract (Supplemental Table S2). Furthermore, we reviewed reference lists of original and review articles to search for more studies.

### 2.2. Study Selection and Data Extraction

The titles and abstracts of the studies retrieved in the searches were screened to identify the studies that met the predefined inclusion criteria. The full texts of the potentially eligible studies were then retrieved and assessed for eligibility. The data selection and extraction were independently conducted and reported in accordance with the PRISMA statement [24] by two reviewers (L.D., E.L.F.). Differences were resolved by discussion and consensus.

For inclusion, studies had to fulfil the following criteria: (i) original article; (ii) intervention trial; (iii) involvement of an adult population; (iv) indication of the difference in outcome (i.e., FMD) between the potassium supplementation and control intervention in one or more patient cohorts; and (v) indication of the number of participants included in the control and exposed groups.

The following features of the identified studies and respective populations were recorded: gender, age, country, publication reference, ethnicity, total number of participants, duration of intervention, type of intervention and control, BMI, and potassium and sodium intake.

### 2.3. Risk of Bias

The risk of bias of the studies was evaluated according to the Cochrane risk of bias tool [25].

### 2.4. Grading of Evidence

The quality of the entire body of evidence was assessed by the GRADE (grading of recommendations assessment, development, and evaluation) methodology [26]. Evidence was graded as high, moderate, or low quality. Intervention studies started as high by default. It was downgraded or upgraded based on specified criteria. Criteria to downgrade included study limitations (risk of bias), inconsistency (substantial unexplained heterogeneity), indirectness (factors that limit generalizability), imprecision (95% confidence interval -CI-cross a minimally important difference of 5%), and publication bias (significant evidence of small-study effects). Criteria to upgrade certainty of evidence included a large magnitude of effect, a dose-response gradient, and attenuation by plausible confounding factors.

### 2.5. Statistical Analysis

Mean differences (MDs) and standard errors (SEs) were extracted from the selected publications. If these were not available, MD and SE were calculated from the comparison of the outcomes at high and low potassium regimens. The pooled weighted MD and 95% CI were assessed by a random effect model [27]; *p*-value < 0.05 was considered statistically significant. The influence of the individual cohorts or of a particular study was estimated by sensitivity analysis, omitting one cohort at a time to verify to which extent the inferences depend on a particular study or group of studies. Statistical heterogeneity across the studies was evaluated by the Cochrane Q test (*p*-value < 0.1 was considered statistically significant) and the I^2^ statistic (30% to 60% may represent moderate heterogeneity, 50% to 90% substantial heterogeneity, and 75% to 100% considerable heterogeneity). Funnel plots were constructed and visually assessed for potential bias of the publication [28]. In addition, formal tests (Egger’s and Begg’s tests) were utilised to evaluate possible bias of the publication. In the case of significant funnel plot asymmetry, suggesting a number of possibly “missing” publications, the pooled estimate was recalculated based on the estimated number of “missing” studies and their effect sizes and SEs by the “trim and fill” method. Meta-regression and subgroup analyses were utilised to identify associations between outcome risk and relevant study’s or patients’ features, as potential sources of heterogeneity: age, gender, total participants, body mass index, length of intervention, year of publication, urinary potassium excretion at low and high potassium intake, urinary potassium excretion difference (intervention vs. control), urinary sodium excretion at low and high potassium intake, urinary sodium difference during changes in potassium intake (intervention vs. control), systolic and diastolic blood pressure at high potassium intake, systolic and diastolic difference (intervention vs. control), country of origin, underlying disease status, type of intervention, type of comparator, and study design.

All statistical analyses were carried out utilising the STATA corp. software (version 11.2; College Station, TX, USA).

## 3. Results

From 110 publications identified, six studies met the predefined inclusion criteria. However, one of them was not included since it was based on mixed interventions [17]. Therefore, five studies were used for the analysis [18,19,20,21,22] (Supplemental Figure S1). The main features of the studies included are reported in Table 1.

Overall, the meta-analysis included 332 participants from four countries. All the studies recruited both male and female participants. Two studies provided multiple cohorts including different types of intervention [18,20]. With respect to the comparison of the effects of higher versus lower potassium intake, all studies had a cross-over design and all but one study were randomized controlled trials [22]. Three investigations were double blind [18,20,21], one single blind [19], and in other one blinding was not specified [22]. All studies used 24-h urine collection to estimate potassium and sodium excretion, used as proxies for potassium and sodium intake during supplementation and control intervention. Three studies used placebo capsules as control [18,20,21], one the participants’ usual diet [19] and another one a controlled diet with low potassium intake [22]. FMD was assessed in all studies using a similar validated method, which examines peripheral artery endothelium-dependent dilation in response to an imposed increase in blood flow and shear stress [16]. The length of intervention ranged from 6 days to 6 weeks. The assessment of the “risk of bias” showed that all but one study were at low risk (Supplemental Table S3).

### 3.1. Effect of Potassium Supplementation on Endothelial Function

**Figure 1:**
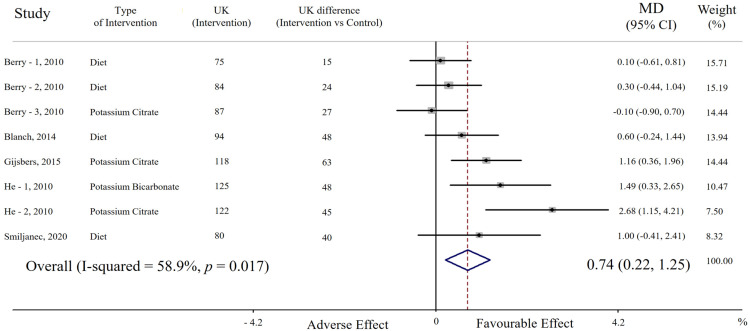
**Forest plot of the effect of potassium supplementation on flow-mediated dilation (FMD).** Results are expressed as mean difference (MD) and 95% confidence intervals (95% CI). Squares indicate study-specific relative risk estimates (size of the square reflects the study-specific statistical weight); horizontal lines indicate 95% CI; diamond indicates the overall relative risk with its 95% CI. UK: urinary potassium excretion (mmol/24 h).

In the pooled analysis including eight cohorts, potassium supplementation (average increase of urinary potassium excretion = 39 mmol/day, range 15 to 63 mmol/day) was associated with significantly higher FMD compared with the lower potassium regimen (MD = 0.74%, 95% CI: 0.22 to 1.25, *p* = 0.005). A moderate heterogeneity among studies was found (*p* = 0.0017; I^2^ = 58.9%) (Figure 1).

A direct association between potassium intake and FMD was detected in seven out of eight cohorts and was statistically significant in three of them; by contrast, there was a non-significant opposite trend in only one cohort (Figure 1). Sensitivity analysis showed that the average change in FMD did not vary substantially with the exclusion of any single cohort or study.

#### 3.1.1. Publication Bias

Visual analysis of the funnel plot showed asymmetry (Supplemental Figure S2), confirmed by formal tests (Begg’s test: *p* = 0.05; Egger’s test: *p* = 0.03). Moreover, the “trim and fill” method identified three possibly missing studies, without substantial changes in the pooled analysis (MD: 0.27%, 95% CI: 0.01 to 0.55).

#### 3.1.2. Additional Analyses

**Figure 2:**
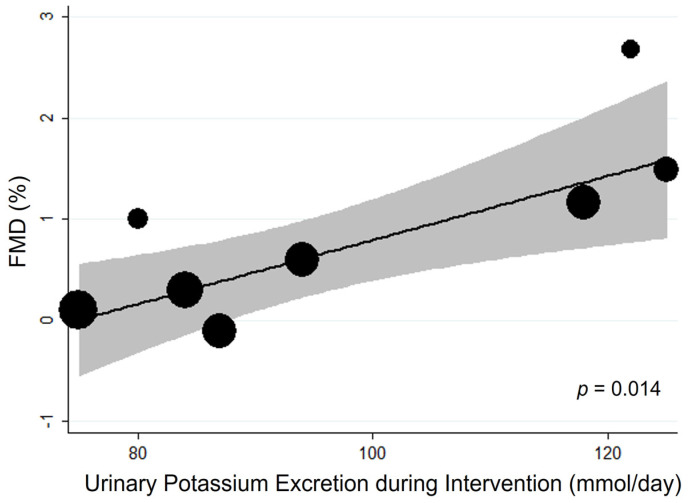
**Bubble plot for random-effects meta-regression of mean difference (MD) in flow-mediated dilation (FMD) by potassium supplementation of the single studies.** Bubbles each represent one study and are plotted according to the study’s MD and urinary potassium excretion of the single study; bubble sizes reflect the relative weight apportioned to studies in the random-effects meta-regression; the solid line indicates the line of best fit. Potassium supplementation is represented by urinary potassium excretion (mmol/day).

Meta-regression analysis detected the amount of potassium supplement as a significant source of heterogeneity (1 mmol higher potassium intake being associated with an increase in FMD of 0.03%, *p* = 0.014) (Figure 2, Supplemental Table S4).

**Figure 3:**
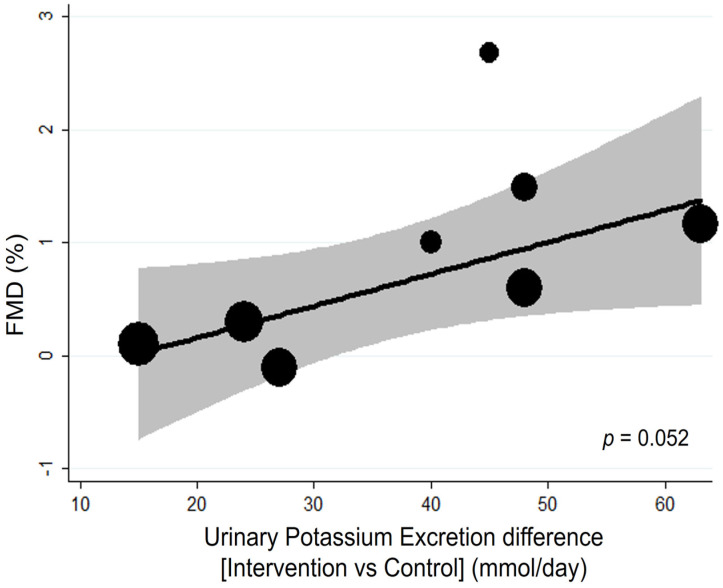
**Bubble plot for random-effects meta-regression of mean difference (MD) in flow-mediated dilation (FMD) by potassium intake difference of the single studies.** Bubbles each represent one study and are plotted according to the study’s MD and urinary potassium excretion of the single study; bubble sizes reflect the relative weight apportioned to studies in the random-effects meta-regression; the solid line indicates the line of best fit. Potassium supplementation is represented by urinary potassium excretion (mmol/day).

The residual variation due to heterogeneity was reduced from 59% to 0%. This result was confirmed by subgroup analysis, in which pooled analysis of the studies with urinary potassium excretion higher than 90 mmol/day showed a significantly greater effect on FMD (MD: 1.30, 95% CI: 0.58 to 2.01) compared with those with excretions lower than 90 mmol/day (*p* for interaction = 0.002) (Supplemental Table S5). Likewise, the difference in potassium intake between intervention and control may also affect the effect of potassium supplements with a borderline significant direct association (*p* = 0.052) (Figure 3, Supplemental Table S4).

Subgroup analysis indicated that the effect of potassium was significant at a urinary sodium excretion lower than 120 mmol/day, but not significantly different from the one detected at higher sodium excretions (*p* for interaction = 0.37) (Supplemental Table S5). This latter result was confirmed by meta-regression analysis, in which the urinary sodium excretion at high and low salt intake and its difference were not significant sources of heterogeneity (Supplemental Table S4). Likewise, blood pressure and its difference during intervention were not identified as sources of heterogeneity (Supplemental Table S4). Meta-regression analysis also suggested no influence of age, gender, total number of participants, BMI, length of intervention, and year of publication on the association between potassium supplement and FMD (Supplemental Table S4). Although the effect of potassium supplementation seemed higher in hypertensive than in healthy participants, the comparison did not indicate a significant difference (*p* for interaction = 0.75) (Supplemental Table S5). The use of capsules for potassium supplementation seemed to have a greater impact on FMD than the dietary approach; however, subgroup analysis did not find a significant difference between the two modalities of intervention (*p* for interaction > 0.05) (Supplemental Table S5). Subgroup analysis also did not reveal country of origin and different type of study design as significant sources of heterogeneity (Supplemental Table S4).

#### 3.1.3. Quality of Body of Evidence

The quality was first downgraded to moderate because of the evidence of publication bias but then it was upgraded again due to the dose–response gradient.

## 4. Discussion

The results of our meta-analysis indicate that supplementation of potassium intake is associated with improvement of endothelial function, expressed as FMD. A positive association was detected between the amount of potassium supplement and its effect on FMD, namely a higher potassium intake being associated with greater FMD increase (i.e., greater vasodilation). In particular, the effect was greater at potassium excretion higher than 90 mmol/day (~4500 mg/day of estimated potassium intake, assuming 1 mmol = 39 mg, and that approximately 70% of potassium ingested is excreted in the urine).

Our results suggest that potassium supplementation could be associated with reduction of cardiovascular risk by improvement of endothelial function. This effect may be translated in healthy people, in patients with cardiovascular risk [16], and in patients with other conditions [29].

It may be hypothesized that the increase in FMD upon the increase in potassium intake is determined by the beneficial effect on blood pressure. However, the analyses show that the peripheral blood pressure at high potassium intake and its difference from control intake did not affect the relationship between potassium intake and FMD.

Furthermore, at relatively low sodium excretion the effect was not significantly better than at higher excretion: it is noteworthy, however, that the urinary sodium excretion (i.e., the level of salt intake) was very high in the majority of the cohorts and this may have attenuated the magnitude of the potassium intake benefit [30].

Our findings are at variance with a previous meta-analysis, in which potassium intake was not associated with improvement of endothelial function [23]. However, the latter study had several limitations, i.e., the inclusion of a smaller number of cohorts, the lack of assessment of possible confounders of the relationship (e.g., the amount of potassium supplement), and assessment of the risk of bias effect in the synthesis of evidence.

A large body of experimental evidence is in support of the association between potassium intake and endothelial function [31]. Particularly, several investigations showed an increase in endothelial nitric oxide production [11] and a suppression of the production of reactive oxygen species after potassium loading, which in turn leads to a significant inhibition of vascular smooth muscle cell proliferation [12,13,14]. The effect of potassium loading was especially evident in the aortic wall of rats, in which a significant decreased lipid peroxide accumulation [32] together with lower endothelial permeability [33] and reduction in macrophage adherence to the vascular wall [34] were detected.

Possible blood-pressure-independent mechanisms were also explored by Rigsby et al. [35], who fed low and high potassium diets to normotensive Wistar Kyoto rats. The main findings of this study showed an improvement of cerebrovascular structure in the high potassium. In addition, in this group at high potassium diet, after experimentally produced cerebral ischemia, there was a significant reduction in the physical size of the resulting cerebral infarct. Furthermore, an increase in potassium concentration in cell cultures increased the nitric oxide release and improved vascular endothelium stiffness [15].

Conceivably, the beneficial effects of high potassium intake may be part of the effects of a Mediterranean diet, typically featuring a large consumption of plant-based foods with a high potassium content. Indeed, a high adherence to the Mediterranean diet has been associated with a reduction of cardiovascular risk [36,37] and an improvement of endothelial function [38].

Our results are in agreement with the previous literature on the relationship between potassium intake and cardiovascular risk factors [1,2,5] and events [3,4].

In particular, one meta-analysis pointed to 90–120 mmol/day as the optimal intake for reducing blood pressure and as associated with a lower risk of incident stroke, while intakes above 120 mmol/day did not seem to have any additional benefit [1]. A more recent meta-analysis on changes in blood pressure showed a U-shaped relationship between potassium intake and blood pressure with a maximum benefit at approximately 90 mmol/die of potassium excretion (used a proxy of potassium intake) and an inverse trend at both lower and higher potassium excretion [2]. Although different design and outcomes were explored, the results of our meta-analysis found a maximum benefit on endothelial function being detected between 90 and 120 mmol/day of urinary potassium excretion, while no data were available to evaluate the effect of a higher intake.

Serum potassium concentration is a poor indicator of dietary potassium intake in the general population. Indeed, hypokalaemia from insufficient dietary potassium intake is rare, while it may be associated with severe hypocaloric diets or occur as a result of an in-creased requirement for the synthesis of tissue during recovery from malnutrition. Like-wise, hyperkalaemia following excessive dietary potassium intake is rare in the general population, being, however, more likely in individuals with impaired renal function [39] or with very high intakes of oral potassium supplements or parenteral potassium administration.

Despite the mechanisms involved in the maintenance of a tight potassium balance, relatively low serum potassium levels have been reported in some countries, including the U.S. [40] It has been hypothesized that the decrease in the potassium content in several U.S. agricultural products due to decreases in crop-available soil potassium and reduction in the application of potassium fertilizers may contribute to the decline in the potassium intake in the U.S. population [40,41]. Furthermore, anthropological and economic changes over the years may have contributed to this deficiency. Indeed, the cost of healthy plant-based foods is more expensive compared with less healthy but energy-dense foods common in Western dietary patterns [42].

Notably, although high potassium intake, and the use of potassium supplementation and of potassium-enriched salt substitutes—at low dose—are safe and do not cause serious untoward side effects such as hyperkalaemia or renal deterioration when provided to individuals or to populations [9,10], and our results show that higher levels of potassium intake were associated with better endothelial function, it would be hazardous to try and reach conclusions on “safe levels” of potassium supplements in addition to dietary intake. Therefore, it seems reasonable to suggest caution with the use of potassium supplementation, particularly if fasting serum potassium concentration is not known [43,44], and in individuals with impaired renal function. On the other hand, the medical prescription of potassium supplementation may be recommended together with a higher dietary intake in case of suboptimal values of serum potassium levels.

As the habitual potassium intake in most countries worldwide is low [6,40,45,46,47,48], an average increase of approximately 40% per day (as found in our meta-analysis) would lead to the achievement of the target of approximately ~4500 mg/day (~90 mmol/day of urinary excretion). Notably, these results also suggest a better effect of potassium supplementation at lower salt intake and, considering the high salt consumption in most countries in the world [45,46,47,49,50], also an associated low salt diet would lead to a reduction of the risk. This observation suggests that the results of our study could be applicable to real-life conditions and are relevant to population-based strategies for an increase in potassium intake, in particular, by fresh fruit and vegetable consumption, coupled with a reduction in salt intake.

Future properly powered controlled trials should be performed to focus on the effect of long-term dietary potassium supplementation on endothelial function, to determine potential cause–effect relationships, to disentangle the effects of possible bias and those of other compounds of potassium rich foods [51,52,53], to assess the “safe levels”, and to overcome the currently limited evidence with respect to the interactions with a number of factors including race, energy needs, and sodium intake [54].

### Study Strengths and Limitations

Major strengths of our meta-analysis are: (i) a trend to increased FMD upon potassium supplementation in most cohorts examined; (ii) the “low risk” of bias in all but one study; (iii) the inclusion of only intervention trials with exclusive evaluation of the potassium effect; (iv) the measurement of 24-h urinary potassium, a recognised gold standard for monitoring potassium [55], in all studies; (v) the inclusion as outcomes of only non-invasively assessed endothelial function; (vi) the direct relationship between the amount of potassium supplementation and the magnitude of the effect; and (vii) the high overall quality of evidence utilising the GRADE assessment approach.

Nevertheless, this meta-analysis has some limitations: first, our results do not allow us to draw definitive conclusions about the long-term effects of supplementation of potassium intake on endothelial function, given that only one trial included in the meta-analysis had an intervention period of 6 weeks.

A second limitation was provided by the small number of the cohorts included and the high heterogeneity of studies’ characteristics. Indeed, additional analyses identified the amount of potassium supplementation as a significant source of heterogeneity, which explained the whole statistical heterogeneity found. Moreover, potassium was provided in different salt formulations and the different anion may be the reason for a different effect. Although the heterogeneity of studies’ characteristics was explored by comprehensive subgroup and meta-regression analyses, which in general did not find significant sources of heterogeneity in addition to the amount of potassium supplement, the tests performed included relatively few studies; hence, definitive conclusions cannot be reached. Third, the detectable publication bias is another limitation; however, the “trim and fill” method identified a number of possibly missing studies and indicated a substantial similar effect of potassium supplementation. Fourth, this meta-analysis was conducted based on aggregated data, thus limiting the possibility to perform additional potential analyses. Fifth, the reproducibility of FMD may be impaired by the occurrence of cardiovascular risk factors [56]. Indeed, a larger variation in high-risk individuals may be due to the lower baseline FMD. Likewise, differences in vascular structure and compliance may also contribute to the higher variability [57]. Nevertheless, several meta-analyses showed a significant reduction of cardiovascular events per increased FMD, both in high- and low-risk populations [16]. Finally, a previous meta-analysis found that the level of potassium excretion was influenced by antihypertensive medications [2]. Nevertheless, our analysis was not affected by this bias as all participants were not on antihypertensive therapy.

## 5. Conclusions

The results of this meta-analysis indicate that supplementation of potassium ameliorates endothelial function, directly dependent on the amount of intake. Furthermore, these findings suggest FMD as a possible additional parameter to clinically evaluate the response to potassium supplementation. In keeping with the previous evidence of a favourable effect of higher potassium intake on cardiovascular risk, the results of our meta-analysis support the promotion of its increased intake in the general population to reduce the risk of cardiovascular diseases and stroke, and to protect against bone loss.

## Figures and Tables

**Table 1:** Characteristics of the studies included in the meta-analysis.

First Author, Year [Ref]	Country	Cohort (n. of Participant) [Ethnicity]	Selected Features of the Study Participants	Intervention	Control	Duration	Age (Year) (Range)	BMI (kg/m^2^)	High vs. Low Potassium Comparison (mmol/24 h) *	Sodium Intake at High and Low Potassium Intake (mmol/24 h) *
Berry, 2010 [18]	UK	48 (23 M, 25 W) [White 29, Black 10, Asian 9]	Diastolic BP > 80 and <100 mmHg	Potassium citrate cp—40 mmol/day)	Placebo cp	6 weeks	45.1 (22–65)	28.4	87 vs. 60	113/106
				Potassium Diet—20 mmol/day					75 vs. 60	116/106
				Potassium Diet—40 mmol/day					84 vs. 60	124/106
Blanch, 2014 [19]	Australia	35 (9 M, 26 W)	Healthy participants	Potassium Diet—150 mmol/day	Usual potassium diet	6 days	31 (18–70)	21.7	94 vs. 46	92/103
Gijsbers, 2015 [20]	The Netherlands	36 (24 M, 12 W) [White]	No smoking, Systolic BP 130–159 mm Hg, no CVD, no diabetes, no treatment.	Potassium chloride cp—72 mmol/day	Placebo cp	4 weeks	65.8 (40–80)	27.2	118 vs. 55	96/105
He, 2009 [21]	UK	42 (30 M, 12 W) [White 29, Black 10, Asian 3]	Systolic BP 140–170 mmHg, Diastolic BP 90–105 mmHg, no treatment.	Potassium chloride cp—64 mmol/day	Placebo cp	4 weeks	51 (18–75)	29.7	122 vs. 77	134/127
				Potassium bicarbonate cp—64 mmol/day					125 vs. 77	129/127
Smiljanec, 2020 [22]	USA	33 (16 M, 17 W)	Salt-resistant Healthy participants	Diet—120 mmol/day of potassium	Diet—65 mmol/day of potassium	1 week	27 (22–45)	24	80 vs. 45	220/240

* by 24-h urinary excretion; BP: blood pressure, cp: capsules, M: men, W: women.

## Data Availability

Not applicable.

## Supplementary Materials

The following supporting information can be downloaded at: https://www.mdpi.com/article/10.3390/nu15040853/s1, Figure S1: Stepwise procedure for selection of the studies; Figure S2: Funnel plot of the effect of potassium supplementation (urinary potassium excretion) on flow-mediated dilation (FMD). MD: mean difference; SE: standard error; Table S1: The PRISMA checklist; Table S2: MEDLINE/PubMed search strategy (without restrictions); Table S3: Risk of bias of the studies included; Table S4: Meta-regression analysis of the effect of potassium supplementation on endothelial function; Table S5: Subgroup analysis of the effect of potassium supplementation on endothelial function.
