# Supplementary material for: Effect of Potassium Supplementation on Endothelial Function: A Systematic Review and Meta-Analysis of Intervention Studies
Source: Nutrients. 2023 Feb 8;15(4):853. doi: 10.3390/nu15040853 (PMC9961878; doi:10.3390/nu15040853)
Supplement: Supplementary file 1 [file nutrients-15-00853-s001.zip › nutrients-2171610-supplementary.pdf]

Figure S1

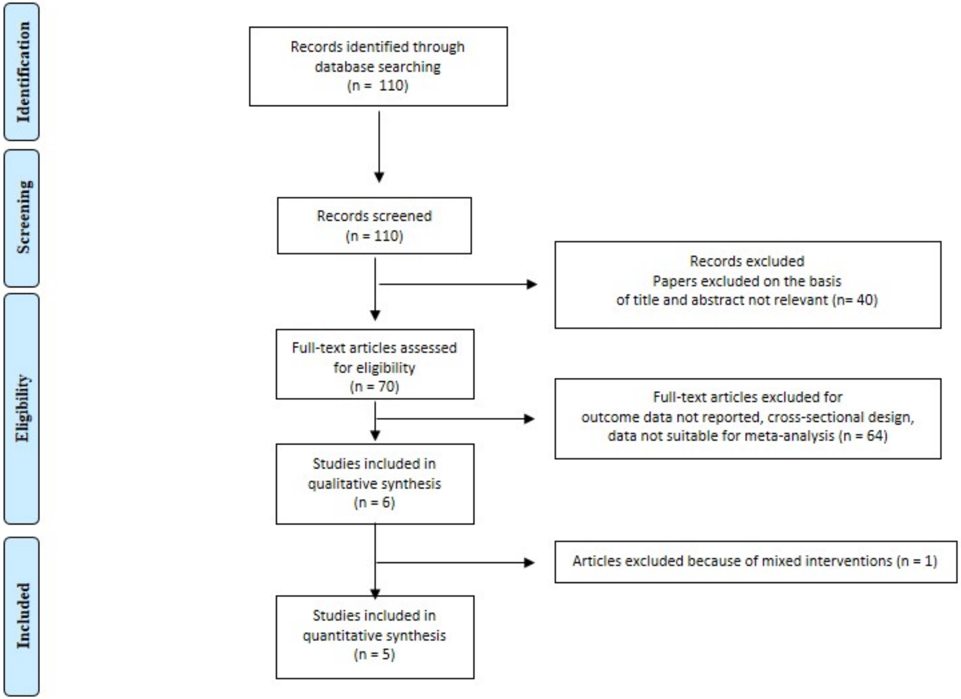
Identification
Screening
Eligibility
Included
Records identified through database searching (n = 110)
Records screened (n = 110)
Records excluded
Papers excluded on the basis of title and abstract not relevant (n= 40)
Full-text articles assessed for eligibility (n = 70)
Full-text articles excluded for outcome data not reported, cross-sectional design, data not suitable for meta-analysis (n = 64)
Studies included in qualitative synthesis (n = 6)
Articles excluded because of mixed interventions (n = 1)
Studies included in quantitative synthesis (n = 5)

Figure S2

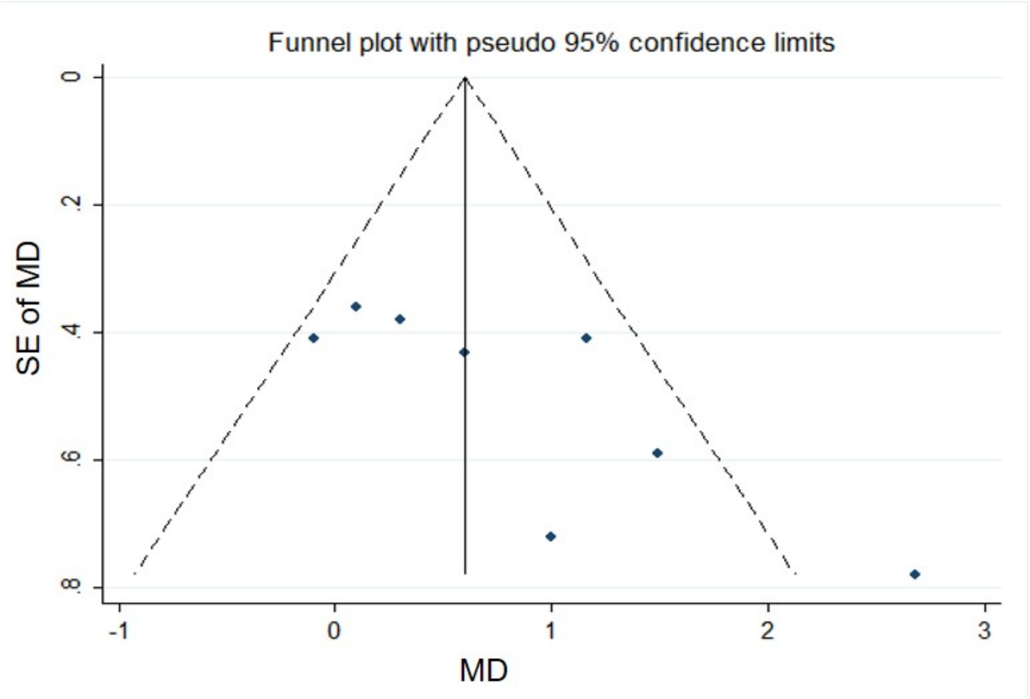

Funnel plot with pseudo 95% confidence limits
0
.2
.4
.6
.8
SE of MD
-1
0
1
2
3
MD

Supplemental Table S1. The PRISMA Checklist

Section/topic	#	Checklist item	Reported on page #
**TITLE**			
Title	1	Identify the report as a systematic review, meta-analysis, or both.	1
**ABSTRACT**			
Structured summary	2	Provide a structured summary including, as applicable: background; objectives; data sources; study eligibility criteria, participants, and interventions; study appraisal and synthesis methods; results; limitations; conclusions and implications of key findings; systematic review registration number.	1
**INTRODUCTION**			
Rationale	3	Describe the rationale for the review in the context of what is already known.	1-2
Objectives	4	Provide an explicit statement of questions being addressed with reference to participants, interventions, comparisons, outcomes, and study design (PICOS).	2
**METHODS**			
Protocol and registration	5	Indicate if a review protocol exists, if and where it can be accessed (e.g., Web address), and, if available, provide registration information including registration number.	2
Eligibility criteria	6	Specify study characteristics (e.g., PICOS, length of follow-up) and report characteristics (e.g., years considered, language, publication status) used as criteria for eligibility, giving rationale.	2-3
Information sources	7	Describe all information sources (e.g., databases with dates of coverage, contact with study authors to identify additional studies) in the search and date last searched.	2
Search	8	Present full electronic search strategy for at least one database, including any limits used, such that it could be repeated.	2
Study selection	9	State the process for selecting studies (i.e., screening, eligibility, included in systematic review, and, if applicable, included in the meta-analysis).	2<br>Figure 1
Data collection process	10	Describe method of data extraction from reports (e.g., piloted forms, independently, in duplicate) and any processes for obtaining and confirming data from investigators.	2-3
Data items	11	List and define all variables for which data were sought (e.g., PICOS, funding sources) and any assumptions and simplifications made.	2-3

Risk of bias in individual studies	12	Describe methods used for assessing risk of bias of individual studies (including specification of whether this was done at the study or outcome level), and how this information is to be used in any data synthesis.	3<br>Supplemental Table S3
Summary measures	13	State the principal summary measures (e.g., risk ratio, difference in means).	3
Synthesis of results	14	Describe the methods of handling data and combining results of studies, if done, including measures of consistency (e.g., I^2) for each meta-analysis.	3
Risk of bias across studies	15	Specify any assessment of risk of bias that may affect the cumulative evidence (e.g., publication bias, selective reporting within studies).	3
Additional analyses	16	Describe methods of additional analyses (e.g., sensitivity or subgroup analyses, meta-regression), if done, indicating which were pre-specified.	3
**RESULTS**			
Study selection	17	Give numbers of studies screened, assessed for eligibility, and included in the review, with reasons for exclusions at each stage, ideally with a flow diagram.	3, Supplemental Figure S1
Study characteristics	18	For each study, present characteristics for which data were extracted (e.g., study size, PICOS, follow-up period) and provide the citations.	4<br>Table 1
Risk of bias within studies	19	Present data on risk of bias of each study and, if available, any outcome level assessment (see item 12).	4<br>Supplemental Table S3
Results of individual studies	20	For all outcomes considered (benefits or harms), present, for each study: (a) simple summary data for each intervention group (b) effect estimates and confidence intervals, ideally with a forest plot.	5<br>Figure 1
Synthesis of results	21	Present results of each meta-analysis done, including confidence intervals and measures of consistency.	5
Risk of bias across studies	22	Present results of any assessment of risk of bias across studies (see Item 15).	5<br>Supplemental Figure S2
Additional analysis	23	Give results of additional analyses, if done (e.g., sensitivity or subgroup analyses, meta-regression [see Item 16]).	5-7

			Suppleme ntal Tables S4-5
**DISCUSSION**			
Summary of evidence	24	Summarize the main findings including the strength of evidence for each main outcome; consider their relevance to key groups (e.g., healthcare providers, users, and policy makers).	8
Limitations	25	Discuss limitations at study and outcome level (e.g., risk of bias), and at review-level (e.g., incomplete retrieval of identified research, reporting bias).	9
Conclusions	26	Provide a general interpretation of the results in the context of other evidence, and implications for future research.	9
**FUNDING**			
Funding	27	Describe sources of funding for the systematic review and other support (e.g., supply of data); role of funders for the systematic review.	10

Supplemental Table S2. MEDLINE/PubMed Search Strategy (without restrictions)

1. dietary potassium
2. potassium intake
3. potassium consumption
4. potassium excretion
5. #1 OR #2 OR #3 OR #4
6. endothelial damage
7. endothelial function
8. flow mediated dilation
9. vascular function
10. #6 OR #7 OR #8 OR #9
11. #5 AND #10

Supplemental Table S3. Risk of Bias of the Studies Included

First author, year [Ref]	Randomization process	Deviation from intended interventions	Missing outcome data	Measurement of the outcome	Selection of the reported result	Overall Bias
Berry, 2010 [18]	Low-risk	Low-risk	Low-risk	Low-risk	Low-risk	Low-risk
Blanch, 2014 [19]	Low-risk	Low-risk	Low-risk	Low-risk	Low-risk	Low-risk
Gijsbers, 2015 [20]	Low-risk	Low-risk	Low-risk	Low-risk	Low-risk	Low-risk
He, 2009 [21]	Low-risk	Low-risk	Low-risk	Low-risk	Low-risk	Low-risk
Smiljanec, 2020 [22]	Some-concerns	Some-concerns	Low-risk	Low-risk	Low-risk	Some-concerns

Supplemental Table S4. Meta-regression analysis of the effect of potassium supplementation on endothelial function.

Variable included in univariate analysis (number of cohorts)	Coefficient (95%Confidence interval)	p-value	I^2-residual (%)
**Age (year) (8)**	.0200702<br>(-.0455541 .0856944)	0.483	60.4
**Gender (men-%) (8)**	.0316012<br>(-.0044173 .0676197)	0.075	41.8
**Number of participants (n) (8)**	-.0632253<br>(-.1659748 .0395242)	0.183	47.1
**BMI (kg/m^2) (8)**	.0438046<br>(-.2460744 .3336837)	0.724	64.8
**Lenght of intervention (day) (8)**	-.0213756<br>(-.0713141 .0285629)	0.335	55.6
**Year of publication (year) (8)**	.0430578<br>(-.195472 .2815877)	0.674	60.1
**Urinary potassium at High potassium intake (mmol/24h) (8)**	.0315564<br>(.0088877 .0542252)	0.014	0
**Urinary potassium at Low potassium intake (mmol/24h) (8)**	.029702<br>(-.0342605 .0936646)	0.299	61.2
**Urinary Potassium difference (intervention vs control, mmol/24h) (8)**	.0282441<br>(-.0003356 .0568239)	0.052	30.4
**Urinary Sodium at High potassium intake (mmol/24h) (8)**	.0047885<br>(-.0178473 .0274243)	0.623	63.9
**Urinary Sodium at Low potassium intake (mmol/24h) (8)**	.0055314<br>(-.0140457 .0251085)	0.515	61.3
**Urinary Sodium difference during changes in potassium intake (intervention vs control, mmol/24h) (8)**	-.0194688<br>(-.081402 .0424645)	0.471	57.4
**Systolic Blood Pressure at High potassium intake (mmHg) (8)**	.011239<br>(-.0529193 .0753973)	0.683	64.7
**Diastolic Blood Pressure at High potassium intake (mmHg) (8)**	.0002305<br>(-.083813 .0842739)	0.995	63.5
**Systolic Blood Pressure difference (intervention vs control, mmHg) (8)**	-.2312228<br>(-.5397356 .07729)	0.116	44.8
**Diastolic Blood Pressure difference (intervention vs control, mmHg) (8)**	-.4641608<br>(-1.054465 .1261429)	0.103	42.7

Supplemental Table S5. Subgroup analysis of the effect of potassium supplementation on endothelial function.

	**Variables (n. of cohorts)**	**MD (95% CI)**	**I^2**	**P for interaction**
**Features of participants**	Healty participants (2)	0.71 (-0.02 to 1.43)	0%	0.75
	Hypertensive participants (6)	0.57 (0.22 to 0.93)	70%	
**Type of Intervention**	Capsule (4)	1.18 (0.14 to 2.22)	76%	0.09
	Diet (4)	0.37 (-0.05 to 0.78)	0%	
**Type of control**	Placebo (6)	0.77 (0.10 to 1.44)	70%	0.85
	Dietary low potassium intake (1)	1.00 (-0.41 to 2.41)	-	
	Usual diet (1)	0.60 (-0.24 to 1.44)	-	
**Study design**	Double-blind (6)	0.77 (0.10 to 1.44)	70%	0.85
	Single-blind (1)	0.60 (-0.24 to 1.44)	-	
	Not blind (1)	1.00 (-0.41 to 2.41)	-	
**Country of origin**	Europe (6)	0.77 (0.10 to 1.44)	70%	0.85
	Oceania (1)	0.60 (-0.24 to 1.44)	-	
	US (1)	1.00 (-0.41 to 2.41)	-	
**Urinary Sodium at High potassium intake** (median= 120 mmol/24h)	< median (4)	0.51 (0.06 to 0.97)	28%	0.37
	> median (4)	1.17 (-0.02 to 2.36)	75%	
**Urinary Potassium at High potassium intake** (median= 90 mmol/24h)	< median (4)	0.19 (-0.23 to 0.60)	0%	0.002
	> median (4)	1.30 (0.58 to 2.01)	48%	
